# Subclinical vitamin A deficiency and associated factors among pregnant women in eastern Ethiopia

**DOI:** 10.3389/fnut.2025.1556074

**Published:** 2025-04-03

**Authors:** Kedir Teji Roba, Gemechu Asefa, Meseret Belete Fite, Abdu Oumer, Dureti Abdurahman, Aboma Motuma, Abebayehu N. Yilma, Gretchen Thompson, Alexandra Brewis, Asher Y. Rosinger

**Affiliations:** ^1^College of Health and Medical Sciences, Haramaya University, Harar, Ethiopia; ^2^Department of Biobehavioral Health, Pennsylvania State University, University Park, PA, United States; ^3^Department of Public Health, Institute of Health Sciences, Wollega University, Nekemte, Ethiopia; ^4^Department of Public Health Sciences, College of Medicine, Pennsylvania State University, Hershey, PA, United States; ^5^Institute of Energy and the Environment, Pennsylvania State University, University Park, PA, United States; ^6^Department of Behavioral, Epidemiological and Clinical Sciences, FHI 360, Durham, NC, United States; ^7^School of Human Evolution and Social Change, Arizona State University, Tempe, AZ, United States; ^8^Department of Anthropology, Pennsylvania State University, University Park, PA, United States

**Keywords:** pregnancy, serum retinol, khat, vitamin A, vitamin A deficiency, Ethiopia

## Abstract

**Introduction:**

Vitamin A is essential for maternal and child health and plays a key role in reducing maternal and child mortality rates. A need exists for more evidence on the prevalence and associated factors of Vitamin A deficiency (VAD) among pregnant women in rural, underserved areas, such as eastern Ethiopia, where many risk factors for VAD may be concentrated.

**Methods:**

A community-based cross-sectional study was conducted with 397 randomly selected pregnant women at the Haramaya University Demographic Health Surveillance sites. Data were collected through structured questionnaires, anthropometric measurements, blood serum samples, and other relevant household and individual-level information. Vitamin A deficiency (VAD) was defined as serum retinol levels <0.7 μmol/L, while marginal deficiency was defined as 0.70–1.05 μmol/L. Bivariable and multivariable logistic regression analyses were used to identify factors associated with VAD.

**Results:**

Approximately 48.1% (43.1–53.1%) of pregnant women in eastern Ethiopia had subclinical vitamin A deficiency (VAD), with a mean serum retinol concentration of 0.82 (±0.02) μmol/L. Only 122 (30.7%) and 159 (40.1%) of the participants reported having adequately diversified diets and adequate food variety scores, respectively. The use of khat (a stimulant) (adjusted odds ratio [AOR] = 1.67; 95% CI: 1.08–2.57) and a lack of awareness regarding vitamin A-rich foods (AOR = 1.67; 95% CI: 1.04–2.68) were found to be positively associated with VAD. Khat chewing was responsible for approximately 40.1% of VAD cases. Additionally, greater educational attainment of the husband (AOR = 0.47; 95% CI: 0.25–0.90) was significantly protective against subclinical VAD.

**Conclusion:**

Almost half of the pregnant women in this eastern Ethiopian sample were found to have subclinical VAD, highlighting the need for nutritional education during antenatal care and community nutrition awareness campaigns by various stakeholders. Context-specific, targeted behavioral change communications are essential to improve dietary practices and healthcare utilization.

## Introduction

Vitamin A is an essential nutrient that is required for normal metabolism, vision, cell function, epithelial integrity, red blood cell production, immunity, growth, and reproduction (1). It is best obtained from eggs, milk, fish, fruits, and vegetables. When these foods are not consumed sufficiently, especially during pregnancy when increased vitamin requirements are required, vitamin A deficiency (VAD) is likely to develop (2, 3). VAD is already identified as a serious public health issue, particularly in lower-income countries (4, 5). According to the World Health Organization (WHO), VAD affects up to 190 million preschool children and more than 19 million pregnant women (6) mainly due to repeated infections, demand during pregnancy for the mother and developing fetus, and inadequate access to and consumption of vitamin A-rich foods (5, 7, 8).

Moreover, pregnant women have an increased vulnerability to adverse consequences of VAD, mainly due to increased demand for the developing baby, maternal vitamin A need, consumption of poorly diversified diets, and limited access to animal source foods (1, 9–11). The consequences of VAD are severe. Prior studies demonstrate that prenatal VAD could result in increased risk of anemia, low birth weight, preterm delivery, poor infant growth (6, 12), intrauterine growth retardation, preeclampsia/eclampsia, infection, vertical HIV transmission, neonatal and infant mortality, and maternal mortality (13–15). This situation is worsened among pregnant women in lower socioeconomic classes and rural areas where the aforementioned risk factors are widespread. Moreover, chronic malabsorption disorders including celiac disease and Crohn’s disease, impair the body’s ability to absorb vitamin A and other essential nutrients which could increase the risk of VAD (16, 17). This could also further be aggravated by poor sanitation and high burden of intestinal worms leading to malabsorption induced VAD (18).

Due to the interaction of infectious diseases and poor or inadequate diets, Ethiopia has one of the highest rates of macro-and micronutrient deficiencies in Sub-Saharan Africa (SSA) (7, 19–22). Low agricultural output, deeply entrenched food habits, food insecurity and repeated droughts and famines all play a key role for prevailing risk of micronutrient deficiency. For instance, studies conducted over several decades in Ethiopia demonstrated the public-health toll of VAD (1, 4, 23–25), despite various intervention measures implemented since 1989 (6, 8, 25).

The implementation of vitamin A supplementation has the potential to address VAD and reduce its adverse consequences (19). However, there is no vitamin A supplementation coverage for pregnant women and lactating mothers in Ethiopia, and the consumption of vitamin A-rich foods is unevenly distributed (11). On the other side, the popularity of khat chewing (a stimulant plant grown and consumed as a substance commonly in this study area) could aggravate poor nutritional status through various ways. For instance, frequent khat use has been linked to poor appetite, nutrition and limit essential vitamins intake further increasing susceptibility to essential nutrient deficiencies, including vitamin A (11, 26–29). Moreover, increased consumption of refined industrial processed foods (white rice, macaroni, pasta and others), deficient in vitamins (30, 31), further complicates the situation in eastern Ethiopia.

However, as the majority of VAD studies have focused on preschool children, a gap exists understanding the full extent of the problem among pregnant women. Studies are also predominantly based on clinical assessments (physical signs of vitamin A deficiency) (4, 30, 31) rather than biochemical parameters, which tend to underestimate the problem (1).

This study was conducted in eastern Ethiopia, a low-income country with a significant burden of maternal undernutrition and context-specific factors exacerbating VAD. Additionally, there is a lack of comprehensive understanding of dietary practices and healthcare utilization patterns in relation to VAD in this specific context. Previous evidence has emphasized the need for comprehensive research using reliable methods, as much of the prior research focused on clinical assessments and often neglected pregnant women (6). Although routine maternal vitamin A supplementation is not recommended, the poor dietary practices of pregnant women, particularly the lack of animal-source foods, further increase the risk of VAD (32, 33). Therefore, this research aimed to (1) determine the existing prevalence of VAD among pregnant women in eastern Ethiopia, based on direct serum testing, and (2) test for associated factors which are locally relevant to this population. Based on the literature outlined, we hypothesized that low knowledge about dietary vitamin A would be associated with a greater risk of VAD, a potentially important factor given many women in the study region have limited education and low levels of literacy. Prior work has demonstrated that the prevalence of VAD was significantly higher in the eastern half of Ethiopia than in the rest of the country (34), where khat is more widely grown and chewed, suggesting khat use may be a modifiable factor.

## Materials and methods

### Study settings and period

The current study was conducted in the Haramaya Health Demographic Surveillance and Health Research Centre (HDS-HRC) study zone, which was launched in 2018. The HDS-HRC is located in the Haramaya woreda, 500 km east of Addis Ababa. Haramaya is made up of 33 kebeles (the lowest administrative unit in Ethiopia), and the HDS-HRC encompasses 12 rural kebeles chosen to capture a range of geographical and environmental factors. The HDS-HRC kept track of 2,306 pregnant women. The Haramaya district is characterized by mixed farming, with the stimulant khat (*Catha edulis* Forsk.) serving as the primary cash crop in the area (35). Sorghum, vegetables (cabbage, carrot, kale, and others), maize, and other crops are also produced in the area. Refined cereals in the form of rice, pasta, and other processed foods are also widely purchased for household consumption (27, 36).

### Study design, study period, and population

Data came from a community-based cross-sectional study of pregnant women from January 5 to February 12, 2021. The source population for this study consisted of all 2,306 pregnant women in the demographic and health survey cohort. The target population included currently pregnant women residing in randomly selected households from each kebeles of Haramaya district, also part of the DHS cohort. Pregnancy status was initially ascertained by the DHS fieldworkers and further confirmed by self-report during interviews. Additionally, health extension professionals verified pregnancy status. Inclusion criteria required residency in the selected kebeles for at least 6 months prior to data collection.

### Sample size and sampling techniques

As this was a population-based study, the sample size was estimated from the 2,306 pregnant women followed under demographic and health surveillance. The target sample size of 401 was calculated using a single population proportion formula, considering a 95% confidence interval, a 37.8% prevalence of VAD among pregnant women in the Sidama Zone of South Ethiopia (24), a 5% margin of error, and a 10% non-response rate. The calculations were performed using Epi Info software (StatCalc) under multiple considerations.

The sample frame was obtained from the Demographic and Health Survey database, which included 2,036 pregnant mothers. Study participants were selected randomly from each of the eight selected kebele proportional to the number of pregnant women in each kebele. The sample was selected using a computer-generated lottery method. Pregnant women from the selected households were approached and interviewed after obtaining their consent. A detailed description of the sampling methods and procedures has been given elsewhere (32, 37, 38).

### Data collection methods

The survey data were collected using a structured questionnaire through face-to-face interviews conducted by trained field assistants who speak the Afan Oromo local language. The questionnaire included items on women’s socioeconomic status (wealth index), demographic information, obstetric history, dietary intake and knowledge, and blood sample collection. The husband’s education status was recorded as: cannot read or write, can read and write, elementary school (1–8), and high school and above. During the analysis, the variable was dichotomized into illiterate, referring to those who did not attend any formal education and are unable to read or write, and literate, referring to those who can read and write and/or have attended any level of formal education. Wealth index was captured using a 41-item household asset inventory (41 relevant household assets) adopted from the Demographic and health survey questionnaire. Exploratory factor analysis using the principal component analysis was constructed after checking the assumptions (correlation, sample adequacy, and complex structure) (39). Using the identified principal components, factor score were generated. The derived factor scores were ranked to five quintiles, which indicate a continuum of wealth status from the poorest to the wealthiest.

Pregnant women’s nutritional knowledge was assessed using 16 nutritional knowledge questions, where the 70th percentile was used as a cutoff point to classify nutritional knowledge as “better” and “worse.” Moreover, pregnant mothers’ attitude on pregnancy nutrition (vitamin A) was evaluated using a 12-item Likert scale and a sequential principal component analysis was employed to classify attitude level toward maternal nutrition.

To assess maternal nutrition status, participants’ mid-upper arm circumference (MUAC) was measured using standard procedures (40). The MUAC was measured at the midpoint between the acromion process of the shoulder joint and the olecranon process of the elbow joint using a non-stretchable tape measure. Measurements were taken at least twice, and the average value was recorded. Maternal MUAC cutoff value below 23 cm was used to define maternal undernutrition (41). Inventories of household assets were used to estimate household socioeconomic status which were adapted from the demographic and Health Survey module for Ethiopia (40).

The Minimum Dietary Diversity Score for Women was measured by counting the number of distinct food groups consumed in the past 24 h. Data was collected using the FAO 2016 frequency questionnaire, which includes 10 food groups. Each woman participating in the study was asked to recall all communal dishes consumed both inside and outside the compound during the preceding 24 h. The recall was conducted randomly on either weekdays or weekends, as weekends did not have any particular significance concerning dietary intake in the context of our study. We ensured that atypical days, such as local feasts or celebrations, were not included in the recall. A score of less than five food groups indicates an inadequately diversified diet, while a score of five or more food groups indicates an adequately diversified diet (42, 43). Moreover, a food consumption score (FCS) was assessed using standard scales as per the World Food Program guide. The frequency of consumption of each food group per week was multiplied by the weight assigned for each food group to obtain the FCS. Poor food consumption score was defined when FCS ranged between 0 and 21, while borderline and acceptable FCS were defined as 21.5–35 and >35, respectively (32, 44). The consumption of animal source foods (ASF) was assessed by recording how often pregnant women consumed animal-based foods during a specified reference period. The ASF scores were divided into tertiles, with the highest tertile classified as “high” ASF and the combined lower two tertiles classified as “low” ASF. These are described in more detail in previous publications (32, 37, 38).

The Food Variety Score (FVS) is calculated by summing the number of distinct foods consumed in a day, treating foods with the same ingredients as the same, regardless of cooking methods. The score does not consider the quantity or frequency of consumption (45). The FVS was assessed using 27 food items consumed. The total FVS for each subject was calculated and those with a value above the mean were classified as high FVS and those less than the mean as low FVS (37).

### Blood sample collection, serum extraction, and serum retinol level determination

Five milliliters of venous blood samples were collected from each study participant by an experienced laboratory technologist (SST) and placed in a sterile serum separator tube. After centrifuging, blood serum was stored at −80 degrees Celsius before being transferred to the Ethiopian Public Health Institute for retinol analysis. An immune-turbidimetric assay was used to determine serum hs CRP (reagent CRPHS Ref. 04628918190). For assessment of VAD, serum retinol was determined from prepared blood sample using High Performance Chromatography. The corresponding readings were categorized as VAD (below 0.70 mol/L) and marginal VAD (0.70–1.05 mol/L) according to the WHO reference for serum retinol. Serum retinol measurements below 0.70 μmol L^−1^ were categorized as having VAD (46, 47). Similarly, serum ferritin was determined using the Sandwich electrochemiluminescence principle (reagent ferritin Ref. 03737551190), and folate using the Competition electrochemiluminescence principle (reagent folate Ref. 07559992190). Serum ferritin below a value below 15 μg L^−1^ as iron deficiency while having hemoglobin level below 11 g dl^−1^ and serum ferritin below 15 μg L^−1^ to define iron deficiency anemia (48).

### Data quality assurance

A two-day intensive training with the completed questionnaire was given to the research assistants and supervisors before data collection began. Further, a pretest was administered to 10% of the population in Kersa district, and the necessary amendments to the tool were made accordingly. The quality control tool was used to ensure that the instruments, laboratory reagents, and technical performances were all in working order. The National Reference Laboratory for Clinical Chemistry monitored quality assurance during laboratory analysis (EPHI) and trained and experienced laboratory professionals to follow standard operating procedures (SOPs). Calibration was performed daily following the SOPs to detect any analytical errors and validate the laboratory value. Close supervision and feedback were provided by investigators at a regular interval.

### Data processing and analysis

Data were double-entered into EpiData Version 3.1 software and exported to STATA 17 (College Station, TX) for analysis. First, data were cleaned, coded, and checked for missing information and outliers. All the analysis were done in Stata version 17 while excel version 2021 was used for plotting forest plots.

### Modeling

Stepwise backward binary logistic regression was conducted to identify factors associated with subclinical VAD (<0.70 μmol L^−1^) among pregnant women. The omnibus test and the Hosmer-Lemeshow statistical test were used to determine model improvement with inclusion of additional variables and goodness of fit (*p*-value above 0.05), respectively. To control for potential confounders, all variables with *p*-values of 0.25 in the bivariable logistic regression analysis and other biologically plausible factors were considered in the final multivariable model (49, 50). The strength of association was depicted and presented using adjusted odds ratio (AOR) with a 95% confidence interval. Multicollinearity was evaluated using inflated standard errors and/or higher variance inflation factors (VIF > 10), yet no significant multicollinearity was detected. Statistical significance was set at a *p*-value of 0.05 for two-tailed tests.

### Ethical consideration

The Helsinki Declaration ethical principles for medical research involving human subjects guided this study (51). Ethical approval was obtained from Haramaya University’s Institutional Research Ethics and Review Committee (ref No: IRERC/223/2020). Written informed consent was obtained from each study participant after explaining the detailed study procedure, blood sample collection, analysis, risks, benefits, study purpose, and other relevant information to the respondents. For those who cannot read, the information sheet was read to them and asked to provide fingerprint consent. The blood sample was collected by trained laboratory professional in accordance with the standard operating procedure guideline to ensure maximum patient safety. Confidentiality was strictly maintained for any personal and sensitive data.

## Results

### Socio-demographic characteristics of the study participants

A total of 397 pregnant women agreed to participate, reflecting a target sample response rate of 99%. The mean age of study participants was 25.0 (±5.0) years, with 47.4% of women in the age category of 25–34 years. Almost all study participants were “housewives” (96.2%), while those who worked engaged in selling crops or other trading (3.80%). Regarding the husband’s occupation, almost all men (93.7%) were farmers (Table 1).

**Table 1:** Sociodemographic characteristics of sampled pregnant women in Haramaya district, eastern Ethiopia (*n* = 397).

Variables	Freq. (%)
Age of women in years	
15–24	183 (46.1)
25–34	188 (47.4)
35–49	26 (6.5)
Educational level of women	
Cannot read or write	281 (70.8)
Can read and write	26 (6.60)
Elementary school (1–8)	82 (20.6)
High school and above	8 (2.0)
Educational level of the husband	
Cannot read and write	214 (53.9)
Can read and write	60 (15.1)
Elementary school (1–8)	98 (24.7)
High school and above	25 (6.30)
Occupation of women	
Housewife	382 (96.2)
Merchant	15 (3.8)
Occupational status of husband	
Farmer	369 (93.7)
Merchant	28 (6.3)
Family size	
1–5	303 (76.3)
>5	94 (23.7)

### Dietary practices of pregnant women

In the previous 7 days, only 102 (14.8%) of the study participants reported consuming animal-source foods, and 42 (10.6%) reported consuming dark green vegetables, which are rich in vitamin A. Regarding dietary consumption, 316 (79.6%) had an acceptable FCS, while only 122 (30.7%) and 159 (40.1%) of pregnant women had an adequately diversified diet and high food variety score, respectively (Table 2).

**Table 2:** Consumption of food types and standard dietary scores in the last 7 days by pregnant women (*n* = 397) in eastern Ethiopia, based on self-reports.

Variable	Frequency (%)
Ate animal source foods	
No	295 (74.3)
Yes	102 (25.7)
Ate dark green vegetables	
No	355 (89.4)
Yes	42 (10.6)
Dietary diversity score (> = 5 food groups)	
Low	275 (69.3)
High	122 (30.7)
Food consumption score	
Poor (<21)	22 (5.50)
Borderline (21–35)	59 (14.9)
Acceptable (above 35)	316 (79.6)
Food variety score	
Low	238 (59.9)
High	159 (40.1)

The Food Variety Score (FVS) was classified as high if it was above the mean number of food items consumed, and low if it was below the mean FVS.

### Aim 1: prevalence of VAD and nutritional status

The mean (standard deviation) serum retinol concentration was 0.82 (±0.02) mol/L (95% CI: 0.77–0.87 mol/L). Vitamin A deficiency was found in 48.1% (95% CI: 43.1, 53.1%) of the study population. About 21% (83) of pregnant women had elevated CRP, indicative of inflammation, which could affect the serum retinol measurements. Moreover, 42.6% (169) of pregnant women were diagnosed with undernutrition as they had low MUAC. In addition, we have explored other biomarkers of micronutrients which could affect the risk of VAD. A total of 192 (48.36%), 208 (52.4%) and 108 (27.2%) pregnant women had folate, iron deficiency and iron deficiency anemia, respectively (Table 3).

**Table 3:** Subclinical vitamin A deficiency, C-reactive protein, and nutritional status of pregnant women in eastern part of Ethiopia.

Variables	Category	Number	%
Vitamin A status	Normal (> = 0.7 μmol/L)	206	51.9
	Deficient (<0.7 μmol/L)	191	48.1
CRP	Normal	314	79.1
	Higher than 5 mg/L	83	20.9
MUAC	≥23 cm	228	57.4
	<23 cm	169	42.6

To test our second aim (identify associated factors with VAD), we used stepwise backward binary logistic regression. We identified awareness or knowledge about vitamin A-rich foods, non-consumption of animal source foods, khat chewing, husband’s occupational status, number of children, and husband’s educational level as candidate variables for multivariable analysis. According to the multivariable logistic regression model, there were statistically significant associations between VAD and knowledge about vitamin A-rich foods, khat chewing, and the husband’s educational level below *p* = 0.05.

**Figure 1:**
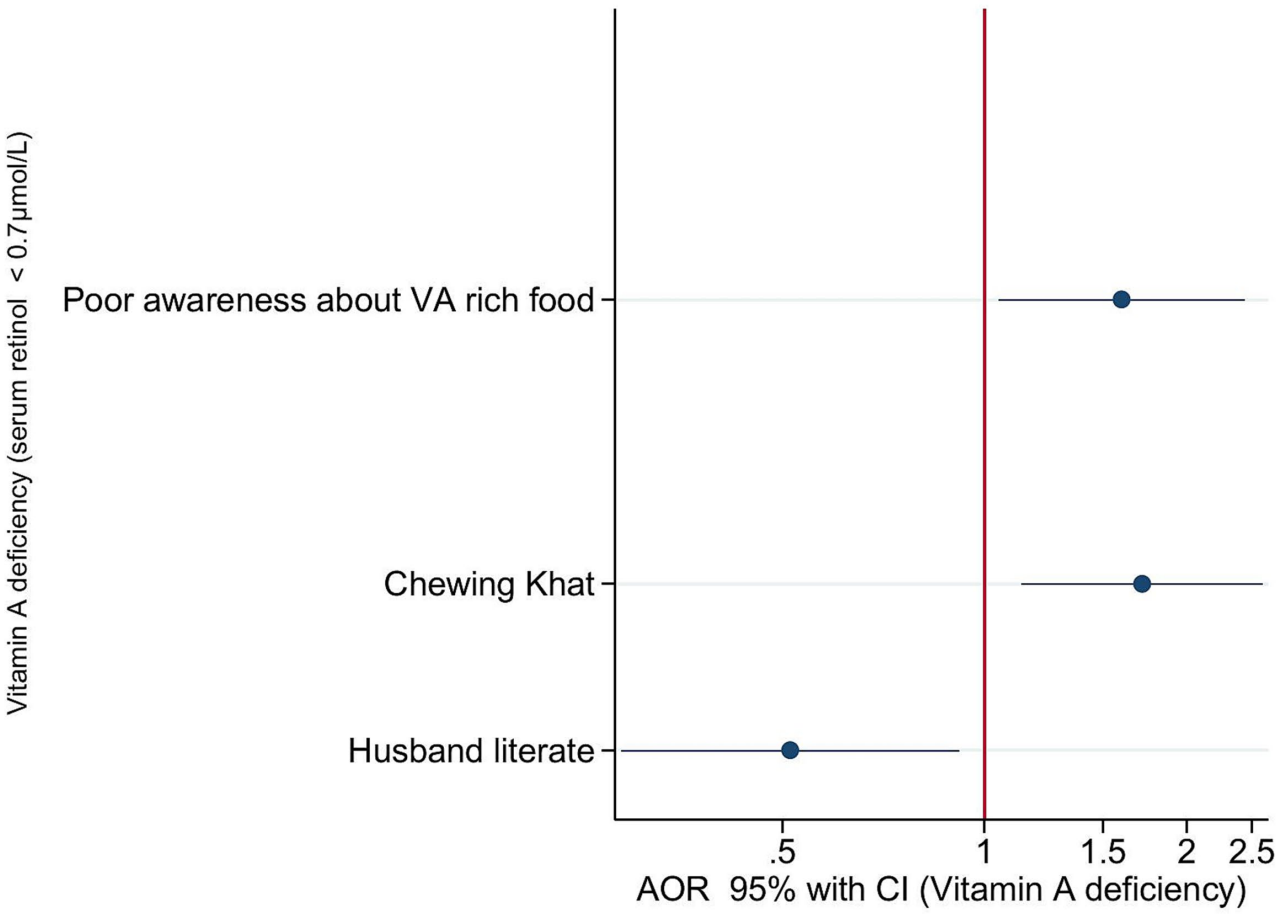
Forest plot showing adjusted odds ratios of statistically significant factors (*p* < 0.05) associated with subclinical VAD (serum retinol <0.7 μmol/L) adjusted for knowledge on vitamin A rich foods, ASF consumption, maternal occupation, khat chewing, and husband educational attainment. In this regression, Animal source food (ASF) consumption, the occupational status of the husband, and parity were included as control variables.

In the fully adjusted model, women who had lower knowledge about vitamin A-rich foods had 67% higher odds of VAD (AOR = 1.67; 95% CI: 1.04, 2.68) compared to those with good knowledge of vitamin A-rich food sources. Similarly, women who reported khat chewing had 67% higher odds of VAD (AOR = 1.67; 95% CI: 1.08–2.57). Compared to women whose husbands were illiterate, women with literate husbands had lower odds of VAD. Specifically, women with literate husbands had 53% lower odds of VAD (AOR = 0.47; 95% CI: 0.25–0.90) compared their counterparts (Figure 1).

## Discussion

This study aimed to understand VAD prevalence and its associated factors among pregnant women in the eastern part of Ethiopia. Overall, we found that nearly half of the pregnant women tested had subclinical VAD. Pregnant women who chewed khat (a stimulant) and lacked awareness about vitamin A-rich foods had higher odds of VAD. In contrast, pregnant women whose husbands attended school had lower odds of subclinical VAD. Approximately 40.1% of the cases of VAD were attributed to khat chewing.

The physiological demands for vitamin A increase during pregnancy, which can lead to adverse health outcomes if these needs are not met. Studies in humans suggest that low or excessive levels of vitamin A in the diet during pregnancy can result in adverse effects on the fetus (52, 72). Maternal VAD is associated with increased risks of complications such as preterm birth, gestational hypertension, and maternal mortality due to infection (53–56). Ahmed et al. (57) reported that pregnant women with VAD had significantly lower hemoglobin concentrations compared to their counterparts with adequate vitamin A levels, indicating a 1.8-fold increased risk of anemia among those deficient in vitamin A. VAD can also affect long-term normal development of the embryo (52, 58) and congenital malformations (59). Additionally, studies suggest that VAD is associated with diabetes mellitus and gestational diabetes (60).

The current study aimed to assess the prevalence of VAD and associated factors in rural eastern Ethiopia. We found that nearly half of tested pregnant women had subclinical VAD. This is higher than previous studies in China (5.3%) (61), Senegal (28.4%) (62), and in Ethiopia (37.9%) (63). Moreover, women’s knowledge of vitamin A-rich foods and chewing khat were associated with greater odds of VAD. A recent food and nutrition strategy baseline survey also indicated that the risk of micronutrient deficiency reached 66% at national levels and 65% in the Oromia region (34). These could be attributed partly to differences in the study area, study period, and socio-cultural conditions, and hence, direct comparison with previous Ethiopian studies is difficult. Variation in actual vitamin A supplementation coverage (64), discrepancies in consumption of vitamin A rich food source and illness patterns may also affect variation in VAD where consumption of extra meal and skipping meals is common (65, 71). These could further frustrate vitamin A intake and could aggravate VAD among pregnant women.

This study found that pregnant women who were less aware of vitamin A-rich foods had a higher risk of developing VAD, consistent with the findings of other Ethiopian studies (11, 63). Greater knowledge of vitamin A-rich foods leads to increased intake of vitamin A-rich foods, and VAD is frequently observed to decrease, especially during pregnancy when nutrient demands increase (10). This could be explained by greater knowledge about vitamin A-rich foods increasing their consumption, as was predicted (58, 59). Antenatal care presents a critical opportunity for nutritional counseling and education, yet studies have shown that many women receive inadequate information regarding their nutritional needs during pregnancy (66, 72).

But a novel primary factor that we found associated with VAD was khat chewing. This is however consistent with previous studies conducted in eastern Ethiopia that have connected khat chewing was associated with greater risk of anemia, suggesting a wider micro-nutrient impact of the practice (36). Elsewhere, khat chewing has been connected to lower ferritin and vitamin B12 in a small sample of male workers (67). The possible pathways between khat chewing and VAD in pregnant women are not well theorized, but could be behavioral, nutritional, and physiological. For one, khat decreases appetite, with a profound effect on the digestive system (26, 68). A review paper suggested that khat chewing is associated with higher odds of undernutrition among adults and the mechanisms tend to be multifactorial (30). Pregnant women are particularly susceptible as they can develop anemia, VAD, and undernutrition simultaneously. Further, the ingredients found in khat like cathine, tannin, and cathinone, appear to influence Vitamin A absorption and catabolism, along with diuretic effects which could also reduce serum retinol (26). Prior work has demonstrated that the prevalence of VAD was significantly higher in the eastern half of Ethiopia than in the rest of the country (34). It is possible that khat chewing played a significant role, given that these activities are uncommon in other parts of the country. However, the mechanisms by which khat causes VAD require further investigation.

The current study also found that women with educated husbands had a protective association with VAD. Our results are consistent with the findings from a study conducted in Bangladesh (69), where better husband’s education was associated with reduced odds of VAD. This may be because men are better educated overall in Ethiopia, and educated partners may better understand the importance of eating a healthy diet during pregnancy and can positively influence their wives to adopt healthy eating habits (10, 24, 70). On the other hand, it could be related to having a better income, self-care, and improved socioeconomic wellbeing that further strengthens the development of healthy behaviors (71). However, the relative influence of these diverse potential mechanisms creating elevated risk of VAD requires further investigation to identify which of these may be the best points of intervention for supporting healthy mothers and babies.

This study has strengths and limitations. This study had a large sample of pregnant women using random sampling to improve generalizability. Second, we used a reliable biomarker of vitamin A status in determining the serum retinol concentrations. The study is also subject to limitations. First, as a cross-sectional design, all relationships should be viewed as associations, and causality cannot be inferred. Second, we identified high CRP in a sub-set of serum samples (20.9%) that could cause some over-estimation of the prevalence of VAD. Additionally, the limited geographical coverage may restrict the generalizability of the study findings beyond eastern Ethiopia. The reliance on maternal self-reports of 24-h dietary diversity and 7-day food consumption scores reflects short-term exposure, whereas serum retinol depletion occurs over a longer period, which was not significant in the regression model. We also anticipated potential recall bias in reporting dietary intake and health behaviors, as well as social desirability bias. However, we made the efforts to mitigate these biases by as training interviewers to ensure accurate reporting and using validated questionnaires, which help reduce the risk of bias in data collection.

## Conclusion and recommendations

Almost half of the pregnant women in this eastern Ethiopian sample were found to have subclinical VAD, highlighting the need for mass nutrition education and awareness campaigns by various stakeholders. Context-specific, targeted behavioral change communications are essential to improve dietary practices and healthcare utilization. Further, we identified that khat chewing and low knowledge of vitamin A-rich foods may be key points of future intervention. Policymakers must prioritize maternal nutrition in public health agendas to mitigate the effects of VAD and improve the overall health of mothers and their children in developing countries. Accordingly, we strongly recommend a robust social and behavioral change intervention program that focuses on encouraging the consumption of vitamin A-rich foods while reducing khat chewing during pregnancy.

## Data Availability

The original contributions presented in the study are included in the article/supplementary material, further inquiries can be directed to the corresponding author.
